# Ultrasound-Assisted Hydrodistillation of Essential Oil from Celery Seeds (*Apium graveolens* L.) and Its Biological and Aroma Profiles

**DOI:** 10.3390/molecules25225322

**Published:** 2020-11-14

**Authors:** Justyna Zorga, Alina Kunicka-Styczyńska, Radosław Gruska, Krzysztof Śmigielski

**Affiliations:** 1 Institute of Natural Products and Cosmetics, Faculty of Biotechnology and Food Sciences, Lodz University of Technology, Stefanowskiego 4/10, 90-924 Lodz, Poland; 2 Institute of Fermentation Technology and Microbiology, Faculty of Biotechnology and Food Sciences, Lodz University of Technology, Wólczańska 171/173, 90-924 Lodz, Poland; alina.kunicka@p.lodz.pl; 3 Institute of Food Technology and Analysis, Faculty of Biotechnology and Food Sciences, Lodz University of Technology, Stefanowskiego 4/10, 90-924 Lodz, Poland; radoslaw.gruska@p.lodz.pl

**Keywords:** optimization, sonication, biological activity, Taguchi method, aroma profile

## Abstract

The aim of the research was to increase the efficiency of the hydrodistillation process and determine the volatile composition, biological activity, and aroma profile of essential oil from celery seeds (*Apium graveolens* L.). The essential oil was extracted from the plant material by ultrasonic hydrodistillation with higher efficiency when compared with classical hydrodistillation. The antimicrobial activity was evaluated using the impedimetric method for the bacteria *Pseudomonas aeruginosa, Escherichia coli, Bacillus subtilis, Staphylococcus aureus,* and yeast *Candida vini* as well as moulds *Aspergillus niger* and *Penicillium expansum* with minimal inhibitory concentration (MIC) (μL/mL) values: 30, 10, 20, 3, 30, 40, and 40, respectively. The oil possessed very weak 2,2-diphenyl-1-picrylhydrazyl (DPPH) antioxidant activity with the half maximal inhibitory concentration (IC_50_) value of 81.6 g/L. Initial studies of the aroma profile indicated that the perception of the fragrance of the oil could be related to the sex of the panellists. According to women, the fragrance of celery seeds oil was intense herb-like. From the men’s point of view, it had a fresh, mossy, and mushroom scent.

## 1. Introduction

Considering the high biological potential of the essential oils, it is advisable to look for new efficient methods for their extraction. In this study, the raw plant material has been treated with ultrasound action towards increasing the efficiency of the hydrodistillation process, which means an increase in the amount of obtained essential oil per unit of weight of the raw material.

The mechanism of extraction of the essential oils by the ultrasound process is based on two main physical phenomena such as the diffusion across the cell walls and membranes and mechanical destruction of plant cell walls by pressure waves and cavitation, and next, rinsing the contents of cells [1]. From an economic standpoint, the application of ultrasound-assisted hydrodistillation of essential oil is beneficial not only on the grounds of more effective extraction, but also saving energy, solvent, and time consumption [2]. Due to the shortening of the exposure time of the raw material to high temperatures, this process avoids the disadvantages of essential oil conventional extraction, such as the formation of the by-product by decomposition of thermolabile and heat-sensitive compounds [3].

The objective of this study was to improve the yield of the essential oil extraction using distillation assisted by sonication of raw material. There is no information in the literature about the effect of sonication of celery seeds (*Apium graveolens* L., *Apiaceae* Lindl.) on the hydrodistillation of essential oil as well as the obtained product quality. The only research on ultrasound-assisted extraction was conducted by Zor et al. (2017), which was proved as a fast and efficient method for deriving alcoholic extracts from celery seeds, a rich source of antioxidants [4]. The celery and its essential oil are known for their therapeutic, medical, and industrial attributes [5]. Due to antioxidant activities and antimicrobial effects against bacteria, yeast, and moulds, the celery seed oil may be used as alternative natural food preservatives, functional foods, and nutraceutical ingredient [6]. The presented research places special emphasis on environmental action, so the waste celery seeds which did not meet quality criteria and without any sowing value were used.

To maximize the process efficiency, the experiment was planned according to a design of experiment (DOE) approach with the use of the Taguchi method to optimize parameters of the seeds sonication process for savings in time and energy consumption. The qualitative and quantitative composition, physicochemical parameters, biological activity, and aroma profile of the essential oil obtained from raw material sonicated in optimal parameters were analyzed.

The hydrodistillation process, to which sonication of raw material has been applied, is characterized by the high increases in the amount of essential oil per unit of weight of the raw material relative to hydrodistillation. The yield of celery seed essential oil has increased by 48.3%.

## 2. Results and Discussion

### 2.1. Taguchi Experimental Design Approach

The results of the Taguchi optimization process are illustrated in Table 1. At individual applicable working levels, the yield of essential oil was from 1.456 ± 0.011 to 1.978 ± 0.048 g per 100 g of seeds. It was shown that, the higher the Eta value, the higher the efficiency. This is consistent with the assumptions of Taguchi’s loss function the larger the better. According to the analysis of variance (ANOVA), all optimized parameters have a statistically significant effect on the yield of essential oil (*p* < 0.05).

**Figure 1:**
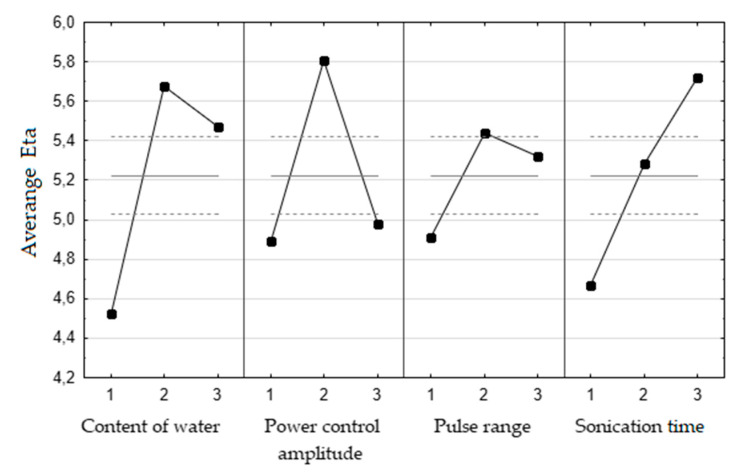
The graphs of average Eta versus input levels of optimized parameters. Solid line—value of Eta, dashed line—±2∙standard error, 1,2,3—input levels for each parameters (content of water: 1–350 mL, 2–700 mL, 3–950 mL; power control amplitude 1–20%, 2–60%, 3–100%); pulse range: 1–0.1, 2–0.5, 3–1; sonication time: 1–5 min, 2–20 min, 3–50 min); *n* = 3.

Initially, the point of inflection on the curve was not obtained in the graph of Eta versus time of sonication (Figure 1). Thus, additional experiments for sonication time (50, 70, and 90 min) under optimum conditions of other parameters were performed. The hydrodistillation efficiency was 2.15 ± 0.032, 2.07 ± 0.06, and 2.04 ± 0.06 g/100 g of seeds, respectively. It was considered that the optimal time of the sonication process was 50 min, due to the lack of significant performance improvement as well as economic and ecological considerations. The optimum conditions for celery seeds pre-treated with the use of ultrasounds were as follows: sonication time 50 min, pulse range 0.5, power control amplitude 60%, and content of water 700 mL (Table 2).

### 2.2. Efficiency of Ultrasonic Hydrodistillation

Statistically calculated expected S/N ratio (Eta) under optimum conditions was 6.971801 (Table 3) which means that, theoretically, under optimal conditions of ultrasound seed treatment, 2.23 g essential oil should be obtained. The experimental yield of essential oil from celery seeds sonicated under optimum conditions equalled 2.15 ± 0.032 g/100 g of seeds. The efficiency increased by 48.3%. The increased efficiency of ultrasound-assisted hydrodistillation results from the specifics of the action of ultrasound [7,8]. The pressure waves and the cavitation led to the mechanical destruction of plant cells, especially cell walls and membranes. The results of this action are the better circulation of extraction solvent, more effective eluting of cell content, and thus more efficient usage of the biological potential of raw material. Another advantage is that sonication accelerates the water absorption leading to faster swelling of the plant material and increasing pore size in the cell walls, and thus facilitates the mass transfer.

According to the literature, celery seed contains approximately 2% of volatile oil [9]. Previous literature studies do not show this method of treatment in the case of hydrodistillation of essential oil from celery seeds. However, ultrasound-assisted extraction is a valuable green and novel technique applied in essential oil hydrodistillation [7]. It has been found that ultrasonic hydrodistillation or maceration significantly affected the increase of the efficiency of essential oil from thyme leaves (*Thymus vulgaris* L.) (about 9%), carrot seeds (*Daucus carota* L.) (about 33%), peppermint leaves (*Mentha piperita* L.) (about 10%), and marjoram herb (*Origanum majorana* L.) (about 12%) [2,10,11].

There was almost a 50% increase in the essential oil efficiency, which confirms the effectiveness of ultrasonic hydrodistillation in the case of extraction of bioactive compounds from hard plant materials like seeds [12]. The process is ecologically significant, because it allows for shortening the distillation stage, electricity, and time consumption. 

### 2.3. Physicochemical Parameters

The physicochemical specification of the essential oil is as follows: density 0.869 ± 0.003 g/mL (20 °C), refractive index $n_{D}^{20}$ 1.47213 ± 0.0004, and optical density ${\left[\alpha \right]}_{D}^{20}$ + 71.08 ± 0.003.

### 2.4. Chemical Composition

**Figure 2:**
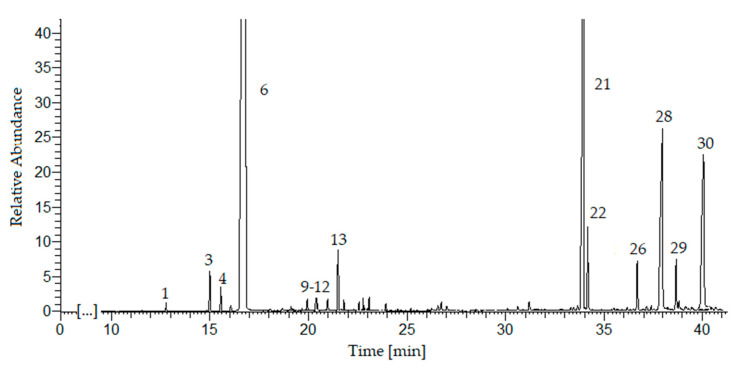
GC-MS chromatogram of the essential oil from sonicated celery seeds (*Apium graveolens* L.). For peak identification, see Table 3.

In the essential oil obtained by ultrasonic hydrodistillation, 30 compounds were identified (Table 3, Figure 2) which constitute 99% of the composition (GC-MS, The NIST Library). The principal chemical constituents were limonene (76.9%), *β*-selinene (9.7%), sedanenolid (3.4%), 3-butylphtalide (3.6%), and *α*-selinene (1.4%), which is consistent with the literature data [14,15,16]. The monoterpenes (78.4%) were the predominant group of chemical compounds, followed by sesquiterpenes (11.1%) and hydrocarbons (7.8%). The monoterpenoids and sesquiterpenoids have been identified in a small amount (total 1.7%).

### 2.5. Similarity Analysis

Statistical analysis consisting of comparing (correlating) individual spectra and determining the correlation coefficient between near-infrared (NIR) spectra of essential oil from celery seeds with and without ultrasounds pre-treatment was performed. The correlation coefficient was 97.8%, which indicates high similarity. The high similarity was also confirmed in the mid-infrared (MIR) spectroscopy analysis, where the specific locations of the bands were identical for both essential oils. According to the Mann–Whitney test with a significant level of 0.05, the amounts of sedanenolide in essential oil obtained by the hydrodistillation as well as from sonicated celery seeds were statistically significantly different [13]. That may be because the sedanenolide, just as some of the volatile phthalides, is unstable due to its active dihydrobenzene structure [17]. Accordingly, decomposition of this compound could have occurred at high temperatures accompanying the hydrodistillation or sonication processes.

### 2.6. Antimicrobial Activity

The essential oil from sonicated celery seeds possessed moderate activity against all tested microorganisms (Table 4). The study demonstrated that the oil had a high antimicrobial effect against food-borne pathogens *S. aureus*. The limonene, pinene (-α, -β), and selinene (-α, -β) are responsible for the biological activity of celery seeds oil [6,18]. The essential oils with a high limonene content like an orange essential oil (*Citrus aurantium dulcis*), a Tahiti lime essential oil (*Citrus limonum*), or a caraway essential oil (*Carum carvi*) inhibited the growth of *S. aureus* with MIC_90%_ values of 16.5, 14.9 mg/mL, and 1.0 μL/mL, respectively [19,20]. Due to virulence and high antimicrobial resistance, *S. aureus* is a common cause of infections and is a serious problem in clinical medicine. Other tested bacteria strains exhibited moderate sensitiveness with MIC values between 10 and 30 μL/mL. Yeast *C. albicans* were more sensitive than moulds and its growth had been inhibited in a concentration of 30 μL/mL. According to Thakre et al. (2018), limonene has destructive effects on the yeast cell surface, thereby resulting in the induction of apoptosis and strongly inhibits *C. albicans* growth [21]. Furthermore, Ünal et al. (2012) indicated that limonene (10 µL) exhibited a higher antifungal activity than antibiotic Fungizone (50 µL) against 12 strains of tested yeast [22]. Celery seed essential oil has been found to exhibit a strong inhibitory effect against *E. coli* and good activity against *P. aeruginosa, B. subtilis*, and *S. aureus* [13,23,24]. There is no marked difference in the biological activity of ultrasound pre-treatment celery seed essential oil when compared with essential oil obtained by classical hydrodistillation [13].

### 2.7. Antioxidant Activity

The essential oil from sonicated celery seeds was characterized by very weak antioxidant activity. Essential oil at the concentrations between 2.5 and 100 g/L expressed an antioxidant effect and quenched the stable free radical DPPH in a range between 34 and 52% (Table 5). The essential oil showed DPPH radical scavenging activity very similar to the control sample [16]. The concentration of essential oil causing 50% inhibition of DPPH radical equalled 81.63 g/L. By way of comparison, the IC_50_ value for vitamin C was 0.0044 g/L, for oregano (*Oregano vulgare* L.) essential oil 0.332–0.501 g/L, and for Chinese fennel (*Foeniculum vulgare* Mill.) essential oil 15.66 mg/g [25,26,27]. In the literature, celery seed essential oil antioxidant activities were defined as weak or moderate [14,28].

### 2.8. Aroma Profile

**Figure 3:**
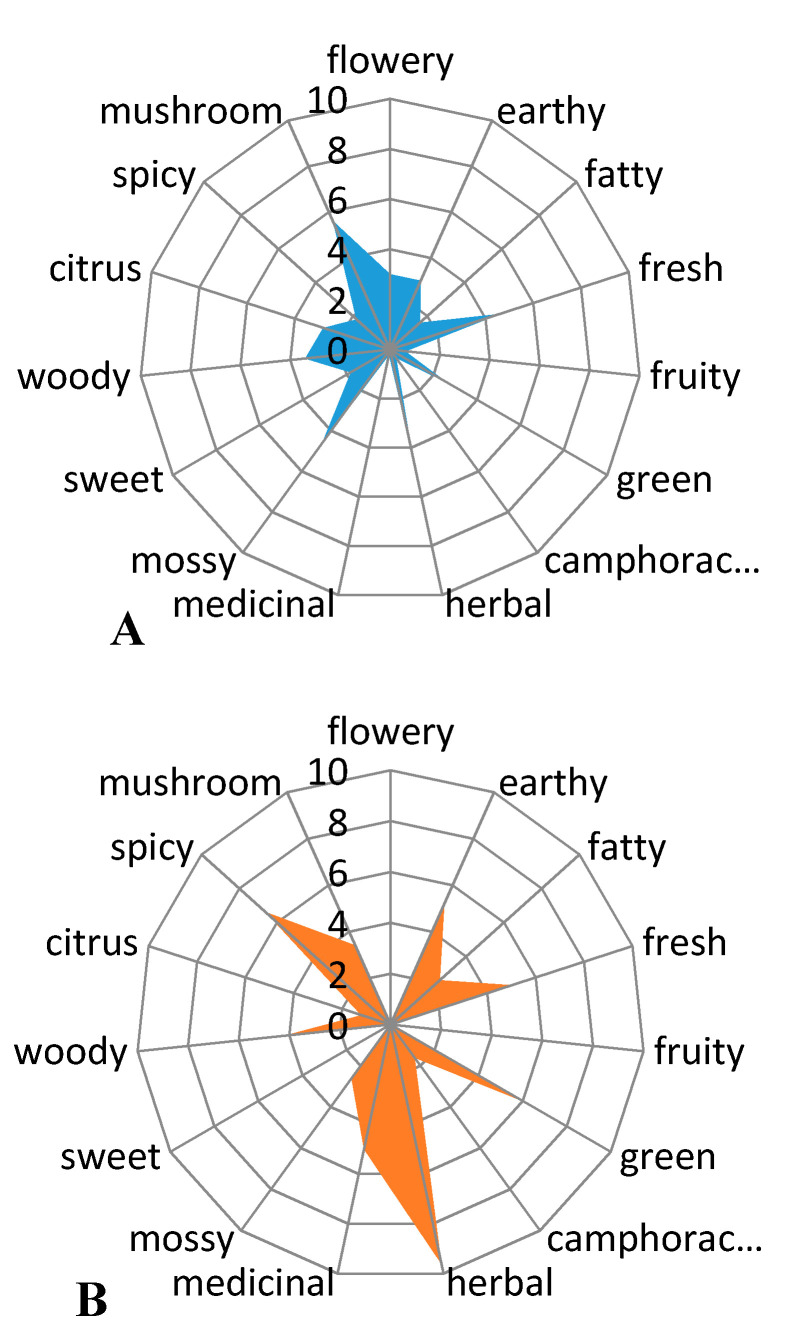
Aroma profile of essential oil from sonicated celery seeds assessed by men (**A**) and by women (**B**) (*n* = 3).

The volatile constituents having a phthalide skeleton: sedanenolide, 3-butylphthalide, sedanolide, and *Z*-ligustilide are responsible for the celery-like aroma of the essential oil and the whole plant [6,9]. The first two of these were identified in the essential oil from sonicated celery seeds. The odor of essential oil was a celery-like, citrus-like, and vegetable-like with noticeable fresh, green notes, and mirepoix scent. Initial studies of the aroma profile indicated that the sensory impressions could be gender-specific (Figure 3), but further analysis of the topic requires more extensive investigation with more panellists.

Women have sensed more scent notes and they have rated the fragrance as more intense than men. According to women, the smell of celery seed oil was intense herb-like, spicy, and green with fresh, earthy, and medicinal notes. Men had a different opinion from women. From their point of view, the celery oil had fresh, mossy, and mushroom scent. It should be noted that women evaluated that the fragrance of oil was herbal and awarded it the maximum number of points, unlike men, who did not award any points there.

It was found that essential oil from sonicated celery seeds possessed slightly intensive fragrance versus the essential oil obtained a classical hydrodistillation [13]. According to panellists, a citrus-like note is slightly more noticeable in essential oil from sonicated seeds, which may be due to a decrease in the content of sedanenolide and an increase in the content of limonene in the chemical composition.

## 3. Materials and Methods

### 3.1. Plant Material

The experimental part of this research was performed with the use of the dried celery seeds (*Apium graveolens* L.) that showed no germination power and were industrial waste with no utility and economic value. Raw material was donated by the manufacturer as part of a cooperation with the Polish seeds company Przedsiębiorstwo Hodowlano-Nasienne W. Legutko Sp. z o.o (Jutrosin). A voucher specimen is stocked in the manufacturer’s premises (Jutrosin, Poland).

The seeds were grounded in laboratory grinders (Basic A11D, IKA, Staufen, Germany) for 20 s.

### 3.2. Taguchi Experimental Design Approach

The experiment has been planned according to a DOE approach with the use of the Taguchi method. The selected control factors and their applicable working levels were: time of sonication 5, 20, 50 min; pulse range 0.1, 0.5, 1.0; power control 20, 60,100 %, as well as the content of water 350, 700, 950 mL. The content of water shall mean the water in the sample treated with ultrasounds. The pulse range was: setting 1 meaning continuously switched on, setting 0.6 meaning power discharge 0.6 s, and pause 0.4 s. In this research, the L9 orthogonal array was used, which has nine rows corresponding to the number of tests, with four columns at three levels. Each level of each parameter has been tested three times, which means that the number of required experiments for this module was 27. In the case of the DOE approach, randomness is desired and should be maintained when possible. In relation to this rule, all the trials and repetitions were unbiased and performed in a completely randomized order. Next, the ANOVA was performed (using the Statistica 13.1 software). The Taguchi’s loss function the larger the better was adopted as the best possible loss function for maximizing product yield. In the case of this function, the best quality standard is infinity, and the higher the actual value (the yield of the essential oil), the better. Based on the analysis of Eta values, the best sets of input parameters have been determined and the optimal parameters of the optimized process have been chosen. Last, run confirmation test with optimum conditions have been done in triple repetition. The essential oil obtained from seeds sonicated in optimal conditions was used for further research as the study sample.

The theoretical yield of essential oil was calculated from the formula for Eta in using loss function: $Eta=-10\cdot \log_{10}[(\frac{1}{n}\cdot \sum \left(\frac{1}{y_{i}^{2}}\right)]\;,$ where *n* is the number of iterations and *y_i_* is the value of the output variable (the essential oil yield).

### 3.3. Application of Ultrasounds

The sonication of 100 g of grounded celery seeds (Seeds Company W. Legutko, Poland) was conducted in sonicator UP400S (400W, 24kHz, Hielschier, Germany) with the sound protection box SB1-16 (Hielschier Ultrasonics, Teltow, Germany), electronic timer for controlling the acoustic irradiation duration (Hielschier, Germany), and the titanium sonotrode Tip H3 type (Hielschier Ultrasonics, Teltow, Germany) for transmitting the ultrasound into the liquid. The application of ultrasound was performed according to the L9 orthogonal array containing applicable working levels for each control parameter according to Table 6. The tests were carried out with care to make sure that the sonotrode was always drowned in the same depth.

### 3.4. Hydrodistillation Process

The extraction of essential oils was performed by the hydrodistillation process with the use of a modified Deryng apparatus [29]. The 1000 mL of distilled water per 100 g of celery seeds was used. The control sample constituted the essential oil from untreated celery seeds (without ultrasound processes). The yield of essential oil was calculated as an average of three hydrodistillation processes. The percentage increase of yield of essential oil was calculated according to the formula: $\frac{Y_{UAH}-Y_{HD}}{Y_{HD}}\cdot 100\%$, where: *Y_UAH_* means the yield of essential oil obtained by ultrasound-assisted hydrodistillation, and *Y_HD_* means the yield of essential oil obtained by hydrodistillation.

### 3.5. Physicochemical Parameters

Physicochemical characteristics have been described with an optical rotation α (a polarimeter Autopol IV, Rudolph Research Analytical, Hackettstown, NJ, USA), a refractive index n_D_^20^ (an automatic refractometer JI57 Donserv, Rudolph Research Analytical, Hackettstown, NJ, USA), and a density (an automatic densitometer DDM2910, Rudolph Research Analytical, Hackettstown, NJ, USA). Each measurement was repeated three times.

### 3.6. Gas Chromatography-Mass Spectrometry with Flame Ionization Detection (GC-FID)

Gas chromatography-mass spectrometry analyses were carried out using a Trace GC Ultra gas chromatograph and a DSQ II mass spectrometer (Thermo Electron Corporation, Beverly, MA, USA) with an Rtx-1 column (length 60 m, internal diameter 0.25 mm, film thickness 0.25 mm, Restek Corporation, Bellefonte, PA, USA). A flow divider (an MS-Column Flow Splitter, SGE Analytical Science, Melrose Park, Australia) collected of signals concurrently from two detectors (FID, MSD, Thermo Fisher Scientific, Waltham, MA, USA). The split ratio was 1:20. The temperature program was from 50 °C (3 min) to 300 °C (30 min), at a gradient of 48 °C (21 min). The temperature of the injector (SSL) and detector (FID) was 280 °C and 300 °C, respectively. The helium flowing at a constant pressure of 200 kPa was used as the carrier gas. The mass spectrometer ionization energy was 70 eV, and the ion source temperature was 200 °C. A full scan was conducted in the mass range from 33 to 420. The analysis was repeated three times. The NIST Library, Wiley 8th edition, and the Adams 4th edition were used.

### 3.7. Similarity Analysis

The essential oil from ultrasound-processed in optimal conditions seeds has been compared in term of chemical composition with the essential oil from celery seeds obtained by hydrodistillation without ultrasound-process. Analysis was performed by the Mann–Whitney test with a significant level of 0.05 and by NIR and MIR spectroscopy.

### 3.8. Near-Infrared (NIR) and Mid-Infrared (MIR) Spectroscopy

Oil samples were scanned in the infrared spectrometer Nicolet iS50 FT-IR (Thermo Fisher Scientific, Waltham, MA, USA) that allows to generate spectra in two ranges: MIR and NIR. MIR analyses were carried out with a DTGS KBr detector, IR light source, and KBr beamsplitter. The range of scans was from 4000 to 400 cm^−1^ with a resolution of 4.00 cm^−1^. The samples were placed in IR Sample Cards (Real Crystal, US) with KBr glass (9.5 mm aperture) and all spectra were accumulated from 32 scans. During NIR measurements there were used: InGaAs detector, white light, and CaF_2_ beamsplitter. The range of scans was from 12,000–4400 cm^−1^ with a resolution of 8.00 cm^−1^. All spectra were accumulated from 32 scans. The samples were placed in the middle of the borosilicate glass tubes (6 × 50 mm) made by Kimble Glass, US. All calculations were made using a commercial analysis software: OMNIC 9.3.30 and TQ Analyst, v. 9.4.45 (Thermo Fisher Scientific, Waltham, MA, USA).

### 3.9. Antimicrobial Activity Assay

The microbes which were originated from the American Type Culture Collection (ATCC) and the Center of Industrial Microorganisms Collection of the Institute of Fermentation Technology and Microbiology, Lodz University of Technology, Poland, WDCM 105 (LOCK) were examined. Strains tested were both Gram-positive (*Bacillus subtilis* ATCC 6633, *Staphylococcus aureus* ATCC 6538) and Gram-negative bacteria (*Escherichia coli* ATCC 8739, *Pseudomonas aeruginosa* ATCC 15442), as well as fungi (the yeast *Candida vini* LOCK 0008, the moulds *Penicillium expansum* LOCK 0535, *Aspergillus niger* LOCK 16404). Microbial strains were sub-cultured on the medium plate count agar (PCA, Merck, Darmstadt, Germany) for bacteria or potato dextrose agar medium (PDA, BTL, Warsaw, Poland) for fungi, and next, activated through double passaging in Tripticase Soy Broth (TSB, Biocorp, Warsaw, Poland) for bacteria or Sabouraud Dextrose Broth (SDB, BTL, Warsaw, Poland) for fungi. Conditions of incubation were 30 °C (*E. coli, B. subtilis*), 37 °C (*S. aureus, P. aeruginosa*) for 24h, and 25 °C for 72 h for fungi. Inoculum of 24-h cultures of each strain was prepared in standard 0.85% sodium chloride solution and adjusted to the final concentration of approximately 107 CFU/mL.

Antimicrobial activity assay was carried out using an impedimetric method (Bactometer M64, bioMerieux, Craponne, France). The celery seed essential oil was diluted in ethyl alcohol (pure P.A., Avantor Performance Materials Polan, Gliwice, Poland) in a ratio of 1:1. Each sample included 0.1 mL of the standardized microorganism inoculum and the essential oil at the examined concentration within the range from 50 to 500 μg/mL. Next, each Bactometer well was filled until a final volume of 1 mL was obtained with the use of the growth medium: general purpose medium (GPM, bioMerieux, Craponne, France) for *B. subtilis, S. aureus*, and *P. aeruginosa*, coliform medium (CM, bioMerieux, Craponne, France) for *E. coli*, and yeast and mould medium (YMM, bioMerieux, Craponne, France) for *A. niger, P. expansum*, and *C. vini*. In the negative controls, instead of the essential oil, 0.5 μg/mL of novobiocin for bacteria or 0.2 μg/mL of cycloheximide for yeast and moulds were added. Positive controls did not contain essential oil, but only 0.1 mL of cell standardized suspension in 0.9 mL of the appropriate medium. The microorganisms were incubated for 72 h at their optimal growth temperatures (30 °C for *E. coli*, *B. subtilis*, 37 °C for *S. aureus, P. aeruginosa*, and 25 °C for fungi). Next, the tested strains were checked for their viability by streaking on the PCA medium. Plates were incubated for 72 h for bacteria and 120 h for fungi at the optimal growth temperatures. The results which were the mean value of three measurements were presented as MIC and MBC.

### 3.10. Antioxidant Activity

Radical scavenging activity was examined using the DPPH assay. First, the 1-μM DPPH (Sigma-Aldrich, Hamburg, Germany) solution in methanol (pure p.a., Chempur, Poland) was prepared. Next, the 200 μL of DPPH was added to 100 μL of the solution of essential oil in methanol at concentrations of 2.5, 5, 10, 20, 50, and 100 g/L, respectively. The analysis was performed using a 96-well polystyrene plate (Nest Biotechnology, Wuxi, China). The samples were incubated in the dark at room temperature for 30 min. As the reference, a methanol solution of Trolox ((±)-6-hydroxy-2,5,7,8-tetramethylchromane-2-carboxylic acid, Sigma-Aldrich, Saint Louis, MO, USA) was used. The absorbance of the DPPH radical was spectrophotometrically analyzed at a wavelength of 517 nm (Modular Multimode Microplate Reader TriStar^2^ S, Berthold Technologies, Oak Ridge, TN, USA). The results were shown as the percentage of inhibition of the DPPH radicals calculated according to the formula: $=\;\frac{\left(A_{0}-A_{1}\right)}{A_{0}}\cdot 100$, where, A_0_ is the absorbance of the control sample (DPPH), and A_1_ is the absorbance of the sample with the essential oil. The results were expressed as the arithmetic mean value of the three consecutive measurements with a standard deviation value.

### 3.11. Aroma Profile

The aroma profile of essential oil was performed by untrained analytical teams of five males and five females. A hedonic ten-point scale test was applied. After inhalation, the panellists marked the intensity scale of aroma from 1 to 10 points, where 0 is none or not perceptible intensiveness, and 10 is strong intensiveness. The distinguishing features as fatty, mossy, green, camphoraceous, herbal, citrus, fruity, earthy, flowery, fresh, medicinal, mushroom, sweet, spicy, and woody were evaluated. Sensory hallmarks were according to El-Zaeddi et al. (2016) with modifications [30]. The results were shown as the average of all measurements.

## 4. Conclusion

The presented research provides important information about the non-conventional method for obtaining essential oils, which is ultrasound-assisted hydrodistillation. This technique provides a high yield of obtained essential oil and with respect for pro-environmental aspects. The product obtained has a chemical composition very similar to essential oil extracted by hydrodistillation. The pre-treatment of the seeds by ultrasounds did not result in a loss in their quality. The essential oil from sonicated celery seeds could be a replacement for ‘traditional’ celery seed essential oil and be used in the cosmetics and food industry as a flavoring agent and adjuvant. In the future, it could substitute for synthetic pesticides and food preservatives.

## Figures and Tables

**Table 1:** Statistical analysis of input factors for ultrasonic pre-treatment of celery seeds according to the Taguchi method.

Test No.	Sonication Time (min)	Pulse Range	Power Control Amplitude (%)	Content of Water (mL)	Essential Oil Yield (g/100 g of Seeds)	Eta
1	5	0.1	20	350	1.456 ± 0.011	3.318555
2	5	0.5	60	700	1.978 ± 0.048	5.922812
3	5	1	100	950	1.731 ± 0.035	4.763080
4	20	0.1	60	950	1.949 ± 0.029	5.797067
5	20	0.5	100	350	1.690 ± 0.011	4.557621
6	20	1	20	700	1.884 ± 0.015	5.501434
7	50	0.1	100	700	1.908 ± 0.061	5.607108
8	50	0.5	20	950	1.961 ± 0.045	5.846586
9	50	1	60	350	1.927 ± 0.043	5.697718

**Table 2:** Expected S/N ratio (Eta) under optimum conditions.

Factor	Level	Effect Size	Standard Error
Contents of water [mL]	2	0.453565	0.097524
Power control amplitude [%]	2	0.582312	0.097524
Pulse range	2	0.218786	0.097524
Sonication time [min]	3	0.493584	0.097524
Expected S/N ratio	6.971801		

**Table 3:** Composition of essential oil from ultrasounds pre-treated celery seeds (GC-MS).

No.	Chemical Compound	RT (min)	RIE	RIL	Area (%)	
					EO_UAH_	EO_HD_ *
1	*α*-pinene	12.8	937	940	0.1	0
2	sabinene	14.4	967	965	tr	0
3	*β*-pinene	15.0	980	972	0.8	0
4	*β*-myrcene	15.5	993	992	0.6	0.1
5	*p*-cymene	16.3	1013	1011	tr	0
6	limonene	16.8	1026	1027	76.9	1
7	*β*-linalool	19.2	1085	1082	tr	0.1
8	octen-1-ol acetate	19.5	1100	1102	tr	0
9	*trans-p-*-mentha-2,8-dien-1-ol	19.9	1103	1103	0.1	0
10	limona ketone	20.0	1106	1105	tr	0
11	*cis-p-*mentha-2,8-dien-1-ol	20.4	1116	1116	0.2	0.1
12	limonene oxide	21.3	1138	1138	0.1	0
13	pentylbenzene	21.5	1144	1146	0.7	−0.1
14	1-pentylcyclohexa-1,3-diene	21.7	1148	1156	0.1	0
15	*p*-mentha-1,8-dien-4-ol	22.1	1167	1174	tr	0
16	*trans-*isocarveol	22.5	1169	1175	tr	0
17	*cis*-dihydrocarvone	22.6	1171	1167	0.1	0
18	*α-*terpineol	22.9	1179	1179	0.1	0
19	dihydrocarveol	23.0	1181	1181	0.1	0
20	*α*-curcumene	23.9	1479	1472	tr	0
21	*β*-selinene (*β*-eudesmene)	34.0	1487	1491	9.7	0.4
22	*α*-selinene (*α*-eudesmene)	34.2	1494	1500	1.4	0.1
23	7-*epi*-*α*-selinene	35.4	1532	1526	tr	0
24	selina-3,7(11)-diene	35.5	1540	1535	tr	0
25	hedycariol	36.1	1545	1541	tr	0
26	*β*-caryophyllene oxide	36.7	1573	1576	0.5	0
27	humulene epoxide 2	37.5	1597	1601	tr	0
28	3-butylphthalide	38.0	1626	1629	3.6	0.5
29	*β*-eudesmol	38.7	1639	1644	0.5	0.1
30	sedanenolide (senkyunolide A)	39.9	1698	1701	3.4 **	−1.1
	total				99.0	
	monoterpenes				78.4	
	monoterpenoids				0.7	
	sesquiterpenes				11.1	
	sesquiterpenoids				1.0	
	other hydrocarbons				7.8	

tr—trace, EO_UAH_—the essential oil obtained by ultrasound-assisted hydrodistillation, EO_HD_—the essential oil obtained by hydrodistillation, RT—retention time, RI_E_ and RI_L_—experimental and literature (the NIST Standard Reference Database Number 69) retention index, *—the difference in the content expressed in percentage point in relation to Dąbrowska et al. (2020) [13], where 0 means no differences in chemical composition, **—a chemical compound that content has changed statistically significantly.

**Table 4:** Antimicrobial activity of essential oil from sonicated celery seeds.

Strain	MIC (μL/mL)	MBC/MFC * (μL/mL)
*Escherichia coli* ATCC 1627	10	70
*Psudomonas aeruginosa* ATCC 1555	30	100
*Bacillus subtilis* ATCC 6633	20	150
*Staphylococcus aureus* ATCC 1803	3	20
*Aspergillus niger* LOCK 16404	40	>300 *
*Penicillum expansum* LOCK 0535	40	>300 *
*Candida vini* LOCK 0008	30	120 *

MIC—minimal inhibitory concentration, MBC—minimal bactericidal concentration, MFC—minimal fungicidal concentration. SD (the standard deviations) equalled 0, (*n* = 3).

**Table 5:** Antioxidant activity of essential oil from sonicated celery seeds.

Concentration of Essential Oil (g/L)	2.5	5.0	10.0	20.0	50.0	100.0
DPPH radicals scavenging effect (%)	34.5 ± 0.3	35.3 ± 0.3	37.9 ± 0.3	39.6 ± 0.3	44.8 ± 0.4	52.9 ± 0.4
Trolox Equivalents (µg/mL)	5.6 ± 0.3	5.8 ± 0.3	6.4 ± 0.3	6.9 ± 0.3	8.2 ± 0.4	10.2 ± 0.4
IC_50_ parameter (g/L)	81.6					

**Table 6:** The experimental layout according to the L9 orthogonal array.

Test No.	Sonication Time (min)	Pulse Range	Power Control Amplitude (%)	Content of Water (mL)
1	5	0.1	20	350
2	5	0.5	60	700
3	5	1	100	950
4	20	0.1	60	950
5	20	0.5	100	350
6	20	1	20	700
7	50	0.1	100	700
8	50	0.5	20	950
9	50	1	60	350
